# The identification value of common echocardiography parameters for improvement of mitral regurgitation after transcatheter aortic valve replacement in patients with aortic stenosis

**DOI:** 10.3389/fcvm.2026.1866427

**Published:** 2026-09-21

**Authors:** Ruifeng Li, Pengjie Gao, Yunlong Ma, Mingjun He, Xiaozhen Zhuo, Shun Wang, Ke Han

**Affiliations:** Department of Cardiology, First Affiliated Hospital of Xi'an Jiaotong University, Xi'an, China

**Keywords:** aortic stenosis, echocardiographic parameters, left atrial-to-ventricular ratio, mitral regurgitation, transcatheter aortic valve replacement

## Abstract

**Objective:**

To analyze the factors influencing mitral regurgitation (MR) improvement in patients with aortic stenosis (AS) following transcatheter aortic valve replacement (TAVR), and to evaluate the identification value of echocardiographic parameters.

**Methods:**

One hundred seventy-eight patients underwent TAVR at the First Affiliated Hospital of Xi'an Jiaotong University were finally enrolled. The severity of MR was graded into four levels based on baseline echocardiographic MR area. MR improvement group was defined as a reduction of at least one grade at 6-month follow-up in patients with grade ≥ 2 at baseline, among whom improvement before discharge was defined as early improvement, with the remainder classified as delayed improvement. Comparative analysis, logistic regression and firth regression were used to analyze factors associated with MR improvement. Receiver operating characteristic (ROC) curve analysis was performed to assess the ability of echocardiographic parameters to identify MR improvement.

**Results:**

Fifty-five patients (30.9%) had MR grade ≥ 2 at baseline, and 46 completed the 6-month follow-up. Comparative analysis revealed that the non-improvement group had lower N-terminal pro-brain natriuretic peptide (NT-proBNP), smaller left ventricular dimensions, and a trend toward larger left atrial diameter. Left atrial-to-ventricular ratio (LAVR) was significantly larger in non-improvement group. Compared with the delayed improvement group, patients with early improvement were younger and had larger left ventricular and atrial diameter. Logistic regression showed that LAVR was independently associated with whether MR improved after TAVR. ROC analysis indicated that LAVR may identify MR improvement after TAVR.

**Conclusion:**

Moderate-to-severe MR could improve after TAVR in majority of patients with severe AS. LAVR, which integrates atrial and ventricular echocardiographic diameters, can provide clues for risk stratification regarding whether MR improves after TAVR.

## 1 Introduction

Aortic stenosis (AS) combined with mitral regurgitation (MR) was the most common combination of multivalvular disease, with approximately one-quarter to one-third of severe AS patients presenting with concomitant MR (1, 2). The traditional surgical approach held that such patients should perform concomitant mitral valve replacement at the time of surgical aortic valve replacement. However, studies have demonstrated that simultaneous surgical replacement of both valves significantly increases the incidence of complications and mortality (3). The development of transcatheter aortic valve replacement (TAVR) and transcatheter mitral valve repair (TMVr) have provided options for a staged therapeutic strategy. Considering most moderate-to-severe MR may improve in AS patients following TAVR, different treatment decisions guiding by reassessment of MR severity after TAVR have become the currently guideline-recommended strategy (4).

Previous study has shown that baseline MR severity before TAVR was associated with the mortality (5), whereas other study suggested that residual MR after TAVR was more closely related to the prognosis (6). Meanwhile, whether MR improves after TAVR and the time required for MR improvement are important for guiding subsequent treatment decisions. However, the time points used to assess MR improvement varied across the existing studies, and findings regarding predictors of MR improvement after TAVR remain inconsistent and controversial.

Above all, this study aimed to analyze the factors influencing MR improvement at different time points in patients with AS following TAVR through a retrospective cohort study, and to evaluate the identification value of echocardiographic parameters.

## 2 Materials and methods

### 2.1 Study population

This study retrospectively enrolled 251 patients who underwent TAVR for aortic valve disease at the First Affiliated Hospital of Xi'an Jiaotong University from January 2016 to December 2023. The inclusion criteria were as follows: (1) meeting the absolute or relative indications for TAVR as specified in the 2025 ESC/EACTS Guidelines for the management of valvular heart disease (7); and (2) undergoing TAVR via a transapical or transfemoral approach. Under the consideration of the progressive expansion of TAVR indications over time, this retrospective application was intentionally adopted according to baseline data to maintain a uniform, contemporary standard across the entire cohort. 178 patients were ultimately included after excluding patients with pure aortic regurgitation (*N* = 52) (8) and those with a history of mitral valve surgery (*N* = 21). The detailed study flowchart was shown in Figure 1.

**Figure 1:**
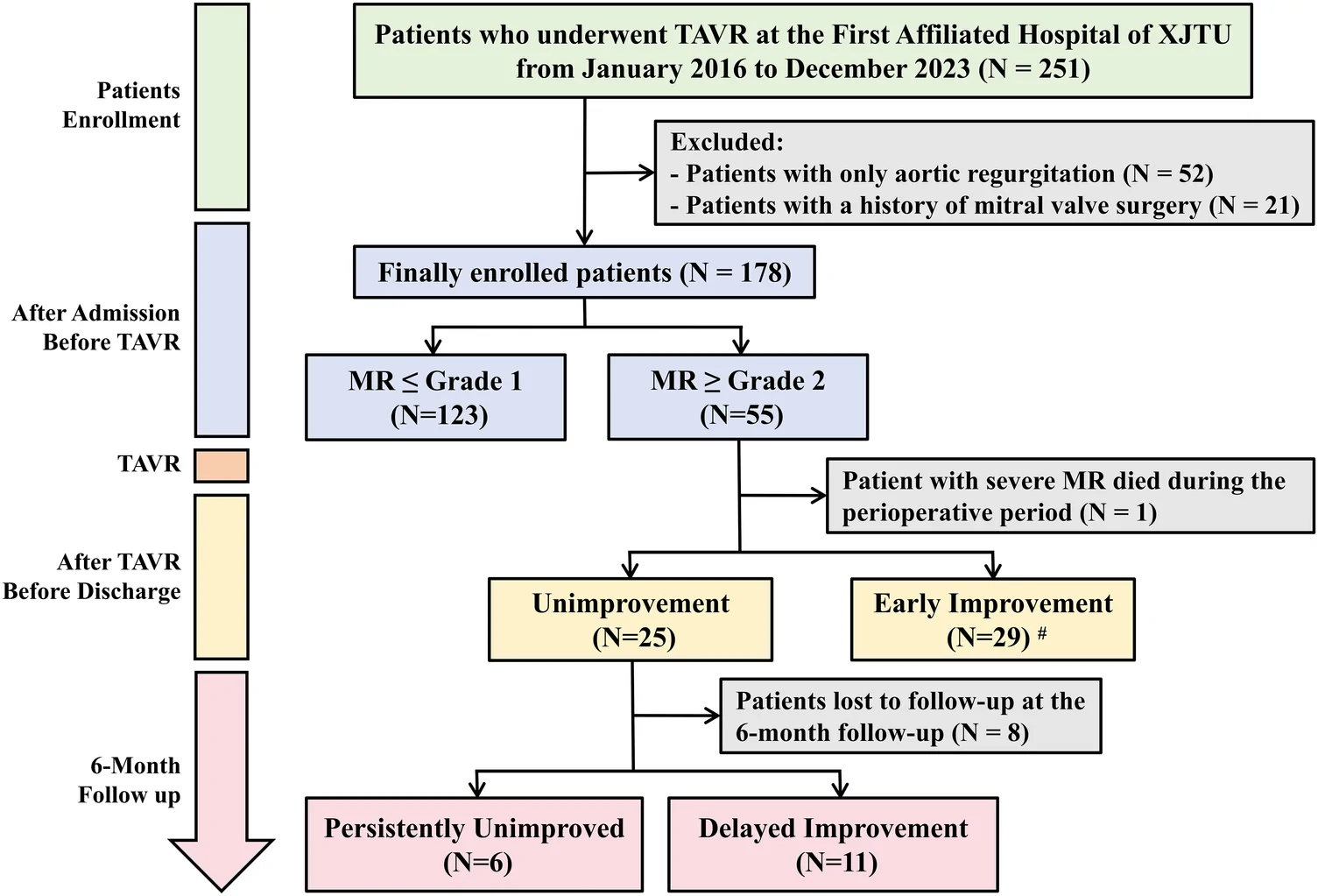
Flowchart of participants selection and grouping. # Among the 29 patients with early MR improvement, 27 had MR of less-than-moderate severity, and 2 had MR severity improved from severe to moderate before discharge. TAVR, transcatheter aortic valve replacement; MR, mitral regurgitation.

This study strictly adhered to the principles of the Declaration of Helsinki and was approved by the Ethics committees of the First Affiliated Hospital of Xi'an Jiaotong University. All patients signed paper informed consent forms.

### 2.2 Echocardiography measurement

Echocardiography (Philips EPIQ 7C, Netherlands) was performed after admission before TAVR, and was repeated before discharge after surgery, 1 and 6 months during follow-up. All images were reassessed by two ultrasonographers blinded to participants’ baseline MR status. The intraclass correlation coefficient was 0.981 [95% confidence interval (95% CI), 0.968–0.988], and the repeatability coefficient was 11.6%.

Echocardiographic parameters were collected including left ventricular end-systolic/end-diastolic anteroposterior diameter (LVESD/LVEDD), interventricular septal thickness (IVST), left ventricular posterior wall thickness (LVPWT), left atrial anteroposterior diameter (LAD), left ventricular ejection fraction (LVEF), aortic valve velocity (AVV), mean aortic gradient, mitral regurgitation (MR) area, aortic regurgitation (AR) area, tricuspid regurgitation area, and estimated pulmonary artery systolic pressure. Relative wall thickness (RWT) was calculated using (IVST + LVPWT)/LVEDD (9); left atrial-ventricular ratio (LAVR) was defined as the ratio of LAD to LVEDD.

The etiology of MR was classified into pathological MR (PMR, defined as the presence of rheumatic changes, or extensive mitral valve prolapse due to mitral valve degeneration, mitral valve prolapse due to chordae tendineae rupture, or mitral valve leaflet calcification combined with mitral insufficiency), atrial functional MR (aFMR, defined as LVEF ≥ 50% without regional wall motion abnormalities; no or mildly dilated left ventricular cavity without leaflet tethering; mitral annulus dilatation; enlarged left atrial), and ventricular functional MR (vFMR, defined as LVEF < 50% with or without regional wall abnormalities; restrictive leaflet motion with tethering; dilated left ventricular dilated) (7).

### 2.3 MR grading and definition of improvement

Based on the preoperative echocardiographic assessment of mitral regurgitation area, the severity of MR was classified as follows: Grade 0 (no MR), Grade 1 (mild, MR area < 4 cm^2^), Grade 2 (moderate, 4 cm^2^ ≤ MR area ≤ 8 cm^2^), and Grade 3 (severe, MR area > 8 cm^2^) (10).

MR improvement was defined as a reduction of at least one grade in regurgitation area among patients with grade 2 or greater MR (11). Patients were divided into two groups according to whether MR improved during the 6-month follow-up. The improvement group was further divided into an early improvement group and a delayed improvement group based on whether MR improvement was observed before discharge after TAVR.

### 2.4 Covariate collection and TAVR procedure

Demographic and medical history information were collected from the medical record system, including gender, age, body mass index (BMI), coronary heart disease (CHD), hypertension, type 2 diabetes mellitus, myocardial infarction (MI), percutaneous coronary intervention (PCI) and atrial fibrillation history. Blood sample was collected at admission and biochemistry test parameters were obtained.

TAVR was performed under general anesthesia via transfemoral or transapical access, with implantation of the aortic valve system in accordance with the ESC/EACTS Guidelines for the management of valvular heart disease corresponding to the respective periods of the procedures (Edition 2012/2017/2021). Postoperatively, all patients received antiplatelet therapy and other medications.

### 2.5 Statistical analysis

Normality of continuous variables was assessed using the Kolmogorov–Smirnov test and frequency distribution histograms. Variables conforming to a normal or approximately normal distribution are expressed as mean ± standard deviation, while non-normally distributed variables are expressed as median (first quartile, third quartile). Comparative analysis was performed using the independent-samples *t*-test for normally distributed continuous variables and Mann–Whitney *U* test for non-normally distributed continuous variables. Categorical variables are expressed as frequency (percentage) and were compared between groups using the chi-square test or Fisher's exact test as appropriate.

The MR improvement rate and regression analyses were based on a complete-case analysis of patients with complete follow-up data (*N* = 46). Logistic regression was used to identify factors associated with whether MR improved following TAVR [Non-improvement of regurgitation was coded as 1 (positive outcome), and improvement was coded as 0 (negative outcome)]. Firth regression and leave-one-out case-deletion analysis were further performed to discuss the stability of the association in small samples. Censored patients during follow-up [perioperative death (*N* = 1) or loss to follow-up (*N* = 8)] were assumed to have no MR improvement, and a sensitivity analysis was performed. Receiver operating characteristic curve (ROC) with the corresponding area under the curve (AUC) was constructed to evaluate the ability of LAVR and related echocardiographic parameters to identify MR improvement following TAVR. All statistical analyses were performed using R (version 4.4.1), and a two-sided *P* < 0.05 was considered statistically significant.

## 3 Results

### 3.1 Baseline characteristics

One hundred seventy-eight patients were finally enrolled, of whom 123 (69.1%) had no significant MR and 55 (30.9%) had moderate-to-severe MR, including 3 patients with significant PMR. Among patients with moderate or greater MR, the proportion of female patients was higher, N-terminal pro-brain natriuretic peptide (NT-proBNP) levels were elevated, LVESD and LAD were larger, IVST was smaller, LVEF was lower, pulmonary artery systolic blood pressure was higher, the proportion of patients with moderate-to-severe AR was greater, and tricuspid regurgitation volume was larger (Table 1).

**Table 1:** Comparison of patients characteristics across groups stratified by severity of MR.

Variables	Total (*N* = 178)	MR ≤ Grade 1 (*N* = 123)	MR ≥ Grade 2 (*N* = 55)	*P* value
Clinical variables				
Age, years	69.1 ± 6.9	68.5 ± 6.6	70.4 ± 7.3	0.084
Male, *n* (%)	103 (57.9)	79 (64.2)	24 (43.6)	**0.010**
BMI, kg/m^2^	23.6 ± 3.6	23.9 ± 3.7	23.1 ± 3.1	0.170
Diabetes, *n* (%)	28 (15.7)	21 (17.1)	7 (12.7)	0.462
Hypertension, *n* (%)	83 (46.6)	61 (49.6)	22 (40.0)	0.236
Prior MI, *n* (%)	5 (2.8)	4 (3.3)	1 (1.8)	0.965
Prior PCI, *n* (%)	9 (5.1)	7 (5.7)	2 (3.6)	0.835
Atrial fibrillation, *n* (%)	23 (12.9)	13 (10.6)	10 (18.2)	0.162
Hemoglobin, g/L	128.5 ± 19.7	129.8 ± 19.8	125.4 ± 19.3	0.164
Ln (NT-proBNP)	7.5 (6.4, 8.3)	7.2 (6.0, 7.9)	8.1 (6.8, 8.8)	**<0** **.** **001**
SCr, μmol/L	78.0 ± 70.8	81.1 ± 82.6	70.9 ± 30.7	0.375
Echocardiographic variables before TAVR				
LVEDD, mm	54.2 ± 9.3	53.1 ± 8.6	56.5 ± 10.5	0.056
LVESD, mm	36.4 ± 9.5	34.8 ± 8.5	39.9 ± 10.6	**0** **.** **001**
IVST, mm	11.8 ± 2.4	12.1 ± 2.5	11.2 ± 1.9	**0** **.** **039**
LAD, mm	38.2 ± 6.9	36.8 ± 6.6	41.3 ± 6.7	**<0** **.** **001**
LVEF, %	65.0 (56.0, 69.0)	65.5 (57.3, 70.0)	62.0 (48.5, 67.0)	**0** **.** **002**
MR area, cm^2^	2.3 (0.0, 4.6)	1.00 (0.0, 2.5)	6.0 (5.0, 8.0)	**<0** **.** **001**
Aortic valve velocity, cm/s	469 ± 82	469 ± 83	472 ± 80	0.808
Mean aortic gradient, mmHg	51.2 ± 19.6	51.1 ± 19.8	51.4 ± 19.5	0.918
Moderate-to-severe AR, *n* (%)	51 (28.7)	29 (23.6)	22 (40.0)	**0** **.** **025**
Pulmonary systolic artery pressure, mmHg	29.9 ± 20.0	25.4 ± 18.8	39.7 ± 19.5	**<0** **.** **001**
TR area, cm^2^	2.0 ± 2.6	1.4 ± 2.4	3.1 ± 2.8	**0** **.** **029**

The bold emphasis indicated that the difference was statistically significant. MR, mitral regurgitation; BMI, body mass index; MI, myocardial infarction; PCI, percutaneous coronary intervention; NT-proBNP, N-terminal pro-brain natriuretic peptide; SCr, serum creatinine; TAVR, transcatheter aortic valve replacement; LVEDD, left ventricular end-diastolic diameter; LVESD, left ventricular end-systolic diameter; IVST, interventricular septal thickness; LAD, left atrial diameter; LVEF, left ventricular ejection fraction; AR, aortic regurgitation; TR, tricuspid regurgitation.

### 3.2 Improvement of MR following TAVR

Among the 178 patients who underwent TAVR, 1 patient with severe MR died during the perioperative period, and 3 patients with mild MR did not undergo echocardiography before discharge. Echocardiography was completed in 174 patients before discharge. The number of patients with less than moderate MR before discharge was 147 (84.5%), while the number of patients with moderate or greater MR was 27 (15.5%) (Figure 2).

**Figure 2:**
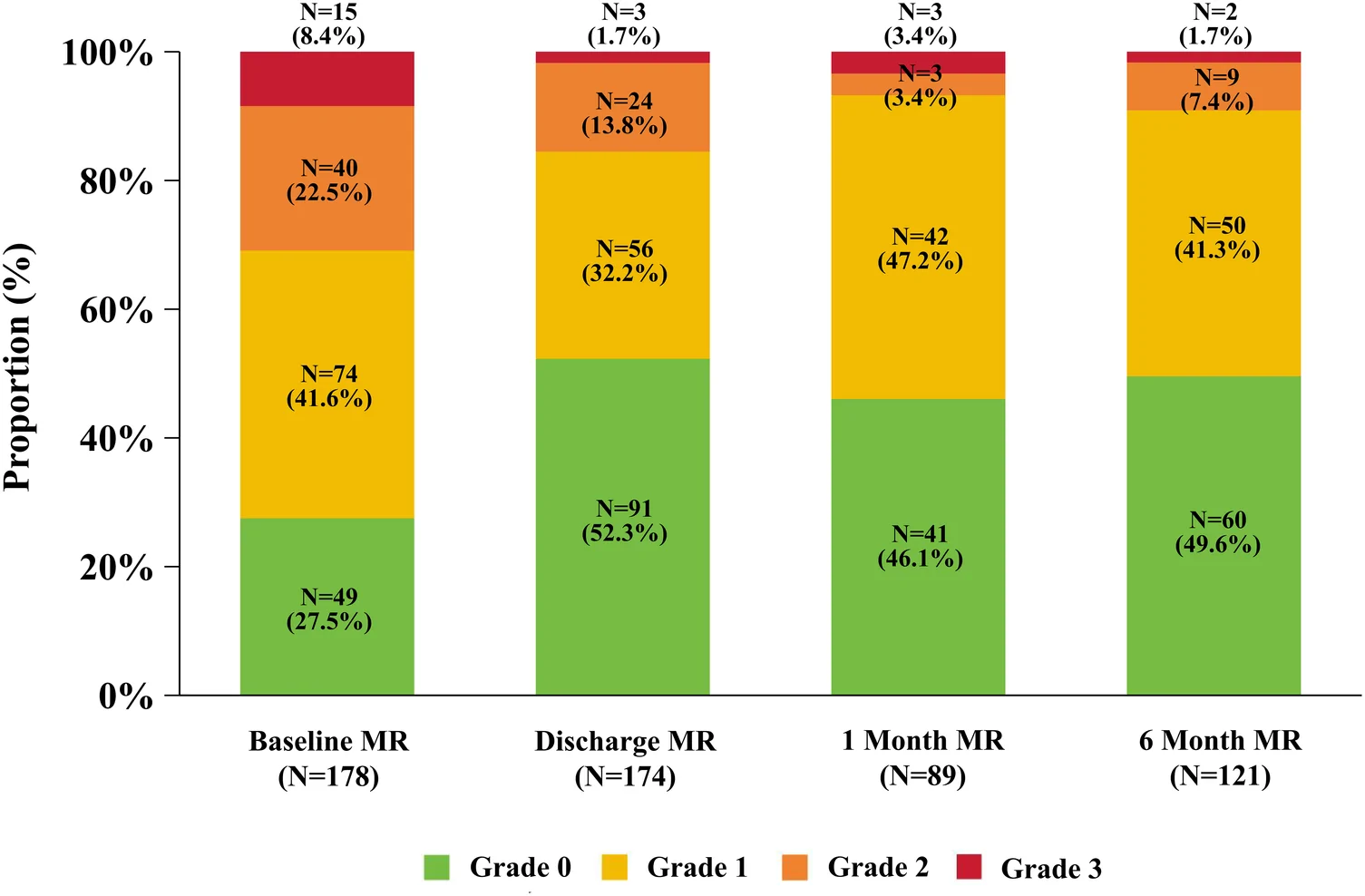
Proportional distribution of MR severity in patients undergoing TAVR at different time points. MR, mitral regurgitation; TAVR, transcatheter aortic valve replacement.

Among the 55 patients with moderate or greater MR at baseline, 46 patients completed 6-month follow-up at our institution. Based on actual follow-up cases alone, early MR improvement rate was 63.1% (29/46), while delayed MR improvement rate was 23.9% (11/46), and MR non-improvement rate was 13.0% (6/46). Under a conservative assumption whereby censored patients were counted as non-improvement, the non-improvement rate was 27.3% (15/55).

### 3.3 Factors influencing MR improvement after TAVR

The 46 patients were divided into improvement group and non-improvement group. Comparison of baseline characteristics revealed that patients in non-improvement group had lower NT-proBNP, smaller LVESD and LVEDD, and a markedly larger LAVR. A trend toward larger LAD was noted in the non-improvement group. Additionally, the non-improvement group showed a trend toward a higher proportion of patients with atrial fibrillation history, though the difference did not reach statistical significance. Comparison of echocardiographic data obtained before discharge revealed that patients in improvement group demonstrated a more pronounced degree of early MR improvement, while no significant difference was observed in the remaining parameters (Table 2). Additionally, baseline comparisons between the 46 patients with complete follow-up and the 9 censored patients revealed no significant differences other than a higher rate of atrial fibrillation in the censored group (Supplementary Table S1).

**Table 2:** Comparison of factors associated with MR improvement during follow-up.

Variables	Improve (*N* = 40)	Not improve (*N* = 6)	*P* value
Clinical variables			
Age, years	70.4 ± 7.4	70.5 ± 7.2	0.963
Male, *n* (%)	20 (50.0)	1 (16.7)	0.276
BMI, kg/m^2^	23.1 ± 2.9	23.9 ± 1.2	0.251
Coronary artery disease, *n* (%)	2 (5.0)	1 (16.7)	0.349
Diabetes, *n* (%)	6 (15.0)	1 (16.7)	1.000
Hypertension, *n* (%)	17 (42.5)	2 (33.3)	1.000
Prior MI, *n* (%)	1 (2.5)	0 (0.0)	1.000
Prior PCI, *n* (%)	2 (5.0)	0 (0.0)	1.000
Atrial fibrillation, *n* (%)	3 (7.5)	2 (33.3)	0.120
Hemoglobin, g/L	124.6 ± 20.8	123.7 ± 16.1	0.915
Ln (NT-proBNP)	8.3 (7.4, 9.1)	6.5 (6.3, 7.1)	**0** **.** **006**
SCr, μmol/L	72.8 ± 35.1	63.2 ± 16.4	0.517
Echocardiographic variables before TAVR			
LVEDD, mm	58.4 ± 8.9	48.5 ± 4.2	**0** **.** **011**
LVESD, mm	41.4 ± 9.8	32.8 ± 3.5	**<0** **.** **001**
IVST, mm	11.4 ± 1.8	9.8 ± 2.1	0.059
LVPWT, mm	10.5 ± 1.9	9.5 ± 1.9	0.263
RWT	0.4 ± 0.1	0.4 ± 0.1	0.775
LAD, mm	40.8 ± 6.2	43.2 ± 6.4	0.381
LAVR	0.7 ± 0.2	0.9 ± 0.1	**0** **.** **006**
LVEF, %	60.0 (47.3, 67.0)	68.0 (65.5, 69.0)	0.099
Aortic valve velocity, cm/s	476.9 ± 87.1	442.5 ± 51.5	0.353
Mean aortic gradient, mmHg	53.4 ± 21.0	44.0 ± 10.9	0.289
MR area, cm^2^	6.0 (5.2, 8.0)	7.3 (5.2, 8.3)	0.648
Type of MR etiology	–	–	0.052
PMR	1 (2.5)	2 (33.3)	–
vFMR	12 (30.0)	1 (16.7)	–
aFMR	27 (67.5)	3 (50.0)	–
AR area, cm^2^	4.9 ± 4.2	2.5 ± 6.1	0.225
Moderate-to-severe AR, *n* (%)	18 (45.0)	1 (16.7)	0.384
TR area, cm^2^	2.8 ± 2.6	2.8 ± 2.8	0.992
Pulmonary systolic artery pressure, mmHg	39.7 ± 20.7	30.5 ± 10.8	0.119
Echocardiographic Variables before discharge			
ΔLVEDD, mm	9.4 ± 9.7	1.8 ± 8.8	0.085
ΔLA, mm	9.0 ± 11.7	−2.4 ± 14.9	0.061
ΔLVEF, %	4.9 (−4.8, 18.1)	2.9 (−9.8, 9.5)	0.744
MR area, cm^2^	2.0 (1.0, 3.7)	5.40 (3.8, 7.8)	**0** **.** **003**
TR area, cm^2^	1.5 ± 1.6	2.7 ± 3.1	0.395
Aortic valve velocity, cm/s	220.6 ± 50.9	220.8 ± 49.1	0.991
Mean aortic gradient, mmHg	11.1 ± 5.3	12.3 ± 8.3	0.615
AVV improvement, %	52.9 ± 10.9	50.2 ± 8.7	0.564

The bold emphasis indicated that the difference was statistically significant. MR, mitral regurgitation; BMI, body mass index; MI, myocardial infarction; PCI, percutaneous coronary intervention; NT-proBNP, N-terminal pro-brain natriuretic peptide; SCr, serum creatinine; TAVR, transcatheter aortic valve replacement; LVEDD, left ventricular end-diastolic diameter; LVESD, left ventricular end-systolic diameter; IVST, interventricular septal thickness; LVPWT, left ventricular posterior wall thickness; RWT, relative wall thickness; LAD, left atrial diameter; LAVR, left atrial-to-ventricular ratio; LVEF, left ventricular ejection fraction; PMR, primary mitral regurgitation; aFMR, atrial functional mitral regurgitation; vFMR, ventricular functional mitral regurgitation; AR, aortic regurgitation; TR, tricuspid regurgitation; AVV, aortic valve velocity.

To identify risk factors independently associated with whether MR improved following TAVR, variables showing significant differences in comparison analysis were included in logistic regression analysis (Table 3). After adjusting for age and sex, LAVR was significantly associated with whether MR improved after TAVR [odds ratio (OR) per 1 standard deviation (SD) = 3.01, 95% CI = 1.06–8.52, *P* = 0.038]. The results indicated that increased LAVR was an independent risk factor for MR non-improvement following TAVR. Further Firth regression and sensitivity analyses indicated that the association between LAVR and whether MR improved after TAVR remained robust and directionally consistent (Supplementary Tables S2, S3). Additionally, the sensitivity analysis classifying censored patients as non-improvement likewise revealed a significant association between LAVR and MR improvement after TAVR (Supplementary Table S4). In contrast, the associations of LVESD and LVEDD with whether MR improved after TAVR were no longer significant after adjustment for confounding factors. Further exploratory regression analysis revealed that the significant association between NT-proBNP and whether MR improvement was attenuated after additional adjustment for BMI, CHD, diabetes and atrial fibrillation history, and LVEF (OR per 1 SD = 0.21, 95% CI = 0.03–1.33, *P* = 0.097), whereas LAVR remained significantly associated with whether MR improvement after TAVR (OR per 1 SD = 4.08, 95% CI = 1.07–15.48, *P* = 0.039).

**Table 3:** Logistic regression analysis of factors influencing MR improvement after TAVR.

Variables	Crude model^a^		Model 1^b^	
	OR (95% CI)	*P* value	OR (95% CI)	*P* value
LAVR (per 1 SD)	3.358 (1.241, 9.087)	**0** **.** **017**	3.009 (1.062, 8.521)	**0** **.** **038**
Ln (NT-proBNP) (per 1 unit)	0.662 (0.451, 0.973)	**0** **.** **036**	0.589 (0.359, 0.967)	**0** **.** **036**
LVEDD (per 1 mm)	0.831 (0.682, 0.982)	**0** **.** **031**	0.818 (0.527, 1.005)	0.050
LVESD (per 1 mm)	0.848 (0.470, 1.037)	0.065	0.823 (0.711, 1.011)	0.083

The bold emphasis indicated that the difference was statistically significant. Non-improvement of regurgitation was coded as 1 (positive outcome), and improvement was coded as 0 (negative outcome). MR, mitral regurgitation; TAVR, transcatheter aortic valve replacement; OR, odds ratio; 95% CI, 95% confidence interval; LAVR, left atrial-to-ventricular ratio; NT-proBNP, N-terminal pro-brain natriuretic peptide; LVEDD, left ventricular end-diastolic diameter; LVESD, left ventricular end-systolic diameter.

a Crude model adjusted for none.

b Model 1 adjusted for age, gender.

Further ROC analysis demonstrated that LAVR could identify MR improvement after TAVR (AUC = 0.83, 95% CI = 0.70–0.97, *P* = 0.009). The AUC of LAVR was larger than that of LVEDD and LVESD, though the Delong test revealed no significant differences in AUC among the three parameters (Figure 3).

**Figure 3:**
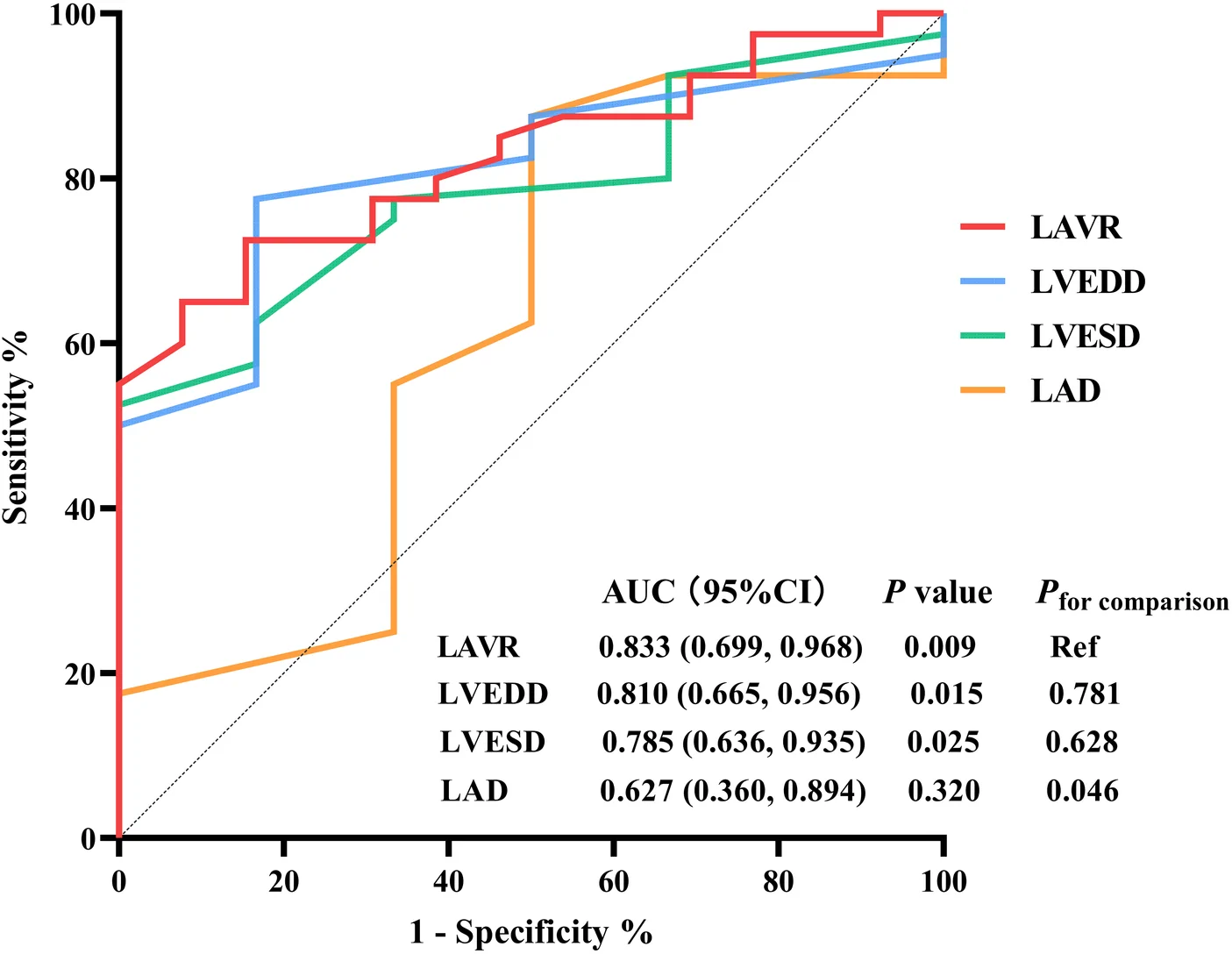
ROC curve of LAVR for identifying MR improvement after TAVR. ROC, receiver operating characteristic; LAVR, left atrial-to-ventricular ratio; LVEDD, left ventricular end-diastolic diameter; LVESD, left ventricular end-systolic diameter; LAD, left atrial diameter; MR, mitral regurgitation; TAVR, transcatheter aortic valve replacement; AUC, area under the curve; 95% CI, 95% confidence interval.

### 3.4 Factors influencing early MR improvement after TAVR

Compared with patients in the delayed MR improvement group, those with early MR improvement were younger, had larger LVEDD and LVESD, larger LAD but no difference in LAVR, lower LVEF, a higher proportion of vFMR, and a lower proportion of aFMR. Early MR improvement was not associated with postoperative AVV, but appeared to be related to the degree of preoperative AR. Specifically, there was a trend toward a higher prevalence of concomitant AR among patients with early MR improvement, although the difference did not reach statistical significance.

Furthermore, compared with the non-improvement group, patients with delayed MR improvement exhibited higher NT-proBNP levels, larger LVEDD, smaller LAD and LAVR (Table 4).

**Table 4:** Comparison of characteristics stratified by improvement or non-improvement of MR at different time points.

Variables	Early improvement (*N* = 29)	Delayed improvement (*N* = 11)	*P* value*	Persistently unimproved (*N* = 6)	*P* value**
Clinical variables					
Age, years	68.5 ± 6.6	75.4 ± 7.2	**0** **.** **007**	70.5 ± 7.2	0.204
Male, *n* (%)	14 (48.3)	6 (54.6)	0.723	1 (16.7)	0.317
BMI, kg/m^2^	23.3 ± 3.0	22.5 ± 2.7	0.413	23.8 ± 1.2	0.239
Coronary artery disease, *n* (%)	1 (3.5)	1 (9.1)	0.479	1 (16.7)	1.000
Diabetes, *n* (%)	4 (13.8)	2 (18.2)	1.000	1 (16.7)	1.000
Hypertension, *n* (%)	13 (44.8)	4 (36.4)	0.900	2 (33.3)	1.000
Prior MI, *n* (%)	0 (0.0)	1 (9.1)	0.275	0 (0.0)	1.000
Prior PCI, *n* (%)	2 (6.9)	0 (0.0)	1.000	0 (0.0)	1.000
Atrial fibrillation, *n* (%)	2 (6.9)	1 (9.1)	1.000	2 (33.3)	0.557
Hemoglobin, g/L	128.3 ± 20.4	114.8 ± 19.3	0.065	123.7 ± 16.1	0.356
Ln (NT-proBNP)	8.5 (7.9, 9.2)	7.64 (7.2, 8.4)	0.787	6.51 (6.3, 7.1)	**0** **.** **013**
SCr, μmol/L	75.9 ± 39.5	64.6 ± 18.2	0.369	63.2 ± 16.4	0.879
Echocardiographic variables before TAVR					
LVEDD, mm	60.1 ± 9.6	54.1 ± 4.8	**0** **.** **014**	48.5 ± 4.2	**0** **.** **032**
LVESD, mm	43.3 ± 10.3	36.4 ± 6.2	**0** **.** **014**	32.8 ± 3.5	0.223
IVST, mm	11.4 ± 1.9	11.5 ± 1.7	0.951	9.8 ± 2.1	0.105
LVPWT, mm	10.3 ± 2.1	11.0 ± 1.6	0.306	9.5 ± 1.9	0.314
RWT	0.4 ± 0.1	0.4 ± 0.1	0.181	0.4 ± 0.1	0.587
LAD, mm	42.2 ± 6.4	36.8 ± 3.7	**0** **.** **012**	43.2 ± 6.4	**0** **.** **019**
LAVR	0.7 ± 0.2	0.7 ± 0.1	0.499	0.9 ± 0.1	**0** **.** **003**
LVEF, %	60.00 (44.0, 66.0)	65.00 (57.5, 69.5)	**0** **.** **035**	68.00 (65.5, 69.0)	0.979
Aortic valve velocity, cm/s	480.5 ± 90.5	467.6 ± 80.7	0.683	442.50 ± 51.47	0.503
Mean aortic gradient, mmHg	53.0 ± 21.7	54.5 ± 19.9	0.851	44.0 ± 10.9	0.255
MR area, cm^2^	6.1 (5.3, 8.0)	5.5 (4.5, 7.7)	0.497	7.3 (5.2, 8.3)	0.185
Type of MR etiology	–	–	**0** **.** **005**	–	0.122
PMR	0 (0.0)	1 (9.1)	–	2 (33.3)	–
vFMR	12 (41.4)	0 (0.0)	–	1 (16.7)	–
aFMR	17 (58.7)	10 (90.9)	–	3 (50.0)	–
AR area, cm^2^	5.2 ± 4.7	3.9 ± 2.5	0.371	2.5 ± 6.1	0.502
Moderate-to-severe AR, *n* (%)	15 (51.7)	3 (27.3)	0.302	1 (16.7)	1.000
TR area, cm^2^	2.9 ± 2.7	2.6 ± 2.4	0.741	2.8 ± 2.8	0.859
Pulmonary systolic artery pressure, mmHg	39.7 ± 20.6	39.7 ± 21.8	>0.900	30.5 ± 10.8	0.351
Echocardiographic variables before discharge					
ΔLVEDD, mm	9.9 ± 11.2	8.6 ± 7.0	0.738	1.8 ± 8.8	0.099
ΔLA, mm	13.0 ± 13.9	5.3 ± 8.4	0.133	−2.4 ± 15.0	0.194
ΔLVEF, %	5.0 (−3.2, 14.6)	1.6 (−9.9, 19.1)	0.854	2.9 (−9.8, 9.5)	0.949
MR area, cm^2^	1.3 (0.0, 2.1)	4.2 (2.9, 5.0)	**<0** **.** **001**	5.4 (3.8, 7.8)	0.169
TR area, cm^2^	1.1 ± 1.3	2.5 ± 2.0	**0** **.** **015**	2.7 ± 3.1	0.866
Aortic valve velocity, cm/s	216.7 ± 51.5	230.9 ± 49.9	0.436	220.8 ± 49.1	0.695
Mean aortic gradient, mmHg	10.6 ± 5.1	12.5 ± 5.7	0.312	12.3 ± 8.3	0.972
AVV improvement, %	54.1 ± 10.6	49.8 ± 11.4	0.266	50.2 ± 8.7	0.938

The bold emphasis indicated that the difference was statistically significant. MR, mitral regurgitation; BMI, body mass index; MI, myocardial infarction; PCI, percutaneous coronary intervention; NT-proBNP, N-terminal pro-brain natriuretic peptide; SCr, serum creatinine; TAVR, transcatheter aortic valve replacement; LVEDD, left ventricular end-diastolic diameter; LVESD, left ventricular end-systolic diameter; IVST, interventricular septal thickness; LVPWT, left ventricular posterior wall thickness; RWT, relative wall thickness; LAD, left atrial diameter; LAVR, left atrial-to-ventricular ratio; LVEF, left ventricular ejection fraction; PMR, primary mitral regurgitation; aFMR, atrial functional mitral regurgitation; vFMR, ventricular functional mitral regurgitation; AR, aortic regurgitation; TR, tricuspid regurgitation; AVV, aortic valve velocity.

* Early improvement group vs. delayed improvement group.

** Delayed improvement group vs. persistently unimproved Group.

## 4 Discussion

This study results showed that most patients with moderate-to-severe MR experienced MR improvement following TAVR. In patients with left atrial enlargement, if ventricular enlargement is not significant, the likelihood of MR improvement is low. However, when concomitant ventricular enlargement, MR may improve early. In patients without significant atrial enlargement but with isolated ventricular enlargement, MR may still improve, but a longer observation period is required. The echocardiographic parameter LAVR can provide clues for risk stratification regarding whether MR improves after TAVR.

Although previous studies have identified numerous predictors for whether MR improved after TAVR, the results remain controversial and inconsistent. This study suggested that identifying potential indicators based on the pathophysiological mechanisms underlying the development, progression and improvement of MR may be a promising direction. Integrating the staging of AS (12), the mechanistic framework for the development of MR in patients with AS mainly includes the following two aspects: (1) Progressive pressure overload: In patients with isolated severe AS, the gradual increase in left ventricular afterload leads to early diastolic dysfunction. The ventricle undergoes concentric hypertrophy to maintain stroke volume, while papillary muscle dysfunction develops and the pressure load is transmitted to the mitral valve, resulting in progressively worsening FMR. MR can improve if the left ventricular pressure load is relieved at this stage. As the disease advances, the increased pressure load is transmitted to the left atrium, causing significant left atrial enlargement, followed by enlargement of the posterior mitral annulus. At this point, MR further worsens and the risk of irreversibility increases. (2) Overlapping factors exacerbating regurgitation: ① If PMR is concomitant, left atrial pressure increase significantly at an early stage, with further enlargement of the LAD. The risk of MR non-improvement after TAVR increases. ② When AR coexists, the increased preload leads to a greater volume of MR. Meanwhile, ventricular dilation occurs under the Frank-Starling mechanism. However, TAVR can relieve both pressure and volume load, thereby improving MR. ③ When ischemic factors such as CHD are present, myocardial remodeling leads to papillary muscle dysfunction and significant MR. Since coronary angiography and PCI are routinely performed alongside TAVR, MR may also improve after ischemia is relieved. In addition, valve type and implantation depth serve as additional factors that influence the degree of pressure load relief, thereby affecting the extent of MR improvement. It is important to emphasize that the mechanisms represent a continuous, dynamic process. Using a simple binary classification may lead to misjudgment of patient status and inappropriate management. Moreover, the presence of overlapping factors in patients increases the difficulty and complexity of identifying predictors for MR improvement after TAVR.

Most previous studies have indicated that significant left atrial enlargement, mitral annular dilation, and pulmonary hypertension are independent risk factors for irreversible MR (13). These factors suggest AS with MR progresses to an advanced stage. At this stage, even though TAVR relieves pressure overload, the risk of MR non-improvement is significantly increased due to irreversible structural damage to the mitral valve itself. Previous literature has also confirmed that PMR exacerbates left atrial and mitral annular dilation, making it an important cause of MR non-improvement after TAVR (1, 11). Atrial fibrillation is also a predictor of MR non-improvement (14), which is consistent with our findings. In most patients with atrial fibrillation, intra-atrial remodeling and fibrosis are present, and left atrial enlargement leads to mitral annular dilation, rendering MR irreversible. Our results show that patients with moderate-to-severe MR have larger ventricular and atrial diameters, higher pulmonary artery systolic pressure, greater tricuspid regurgitation volume. Patients with MR non-improvement had larger LAVR values. These findings are consistent with the mechanistic framework proposed above and with previous literature.

Our findings show that among patients with MR improvement after TAVR, those with larger atrial and ventricular diameters experienced early MR improvement. There was a trend toward a higher proportion of concomitant AR in the early improvement group, while the delayed improvement group had a relatively higher proportion of patients with CHD. Based on the framework proposed above, when patients have increased volume overload, the relative increase in atrial and ventricular diameters may be largely attributed to volume overload, which can recover relatively quickly after TAVR. In contrast, patients with CHD are more prone to ischemia-related ventricular remodeling leading to papillary muscle dysfunction, and MR improvement occurs only after gradual reversal of ventricular remodeling following relief of ischemia and AS (15). Previous literature has also shown that left ventricular enlargement is associated with MR improvement after TAVR, which researchers attribute to concurrent AR leading to ventricular dilation (16).

Furthermore, our results showed that patients with early MR improvement had not yet developed atrial enlargement to the degree observed in the non-improvement group, and their LVPWT and RWT were also not increased to the level of the delayed improvement group. This suggests that pressure overload had not yet become the dominant factor, which is the main reason for early MR improvement. It should be emphasized that our results indicate vFMR tends to improve early, whereas aFMR tends to show delayed improvement. In contrast, previous study has suggested that the proportion of MR improvement was higher in aFMR patients than vFMR (17). This discrepancy may be because those studies did not account for the impact of concomitant AR or low-flow, low-gradient MR on outcomes. Few patients in this study presented with marked eccentric ventricular enlargement accompanied by a significant reduction in LVEF, whom may develop functional decompensation due to multiple factors (such as pressure overload, volume overload, and ischemia). This also explains the conflicting findings in the literature regarding the effect of concomitant AR on MR improvement (18). Future studies should include more such late-stage patients to further validate the conclusions of this study.

Notably, patients with early MR improvement had a larger LAD than those with delayed improvement, suggesting that LAD alone is insufficient to predict MR improvement or its timing. Using the framework described above, we applied LAVR, which integrates atrial and ventricular diameters, to analyze its association with MR improvement after TAVR. The results showed that LAVR was independently associated with MR improvement, and ROC analysis suggested its ability to provide clues for risk stratification regarding whether MR improves after TAVR. Previous literature has shown that LAVR was associated with mortality in patients with hypertrophic cardiomyopathy (19), and the left atrial-to-left ventricular volume ratio has been reported to reflect FMR severity and predict mortality (20). This ratio essentially captures the dominant remodeling pattern between the atrium and ventricle. It should be noted that LAVR was derived solely from LAD and LVEDD in this study due to the high proportion of missing echocardiographic parameters required for volume calculation, whereas the left atrial-to-left ventricular volume ratio used in previous studies may be more powerful in identifying MR improvement after TAVR. Additionally, this study showed LAVR exhibited the largest AUC, which was not significantly different from those of LVEDD and LVESD, but was significantly different from that of LAD. This discrepancy may be explained by the limited ability of LAD diameter alone to accurately reflect left atrial volume and mitral annular area. Previous literature has indicated that the anteroposterior left atrial diameter may have weaker predictive value than other left atrial dimension measurements because it may not reflect the actual width of the mitral annulus. Preoperative cardiac CT-derived left atrial dimension parameters have been shown to be independently associated with MR improvement after TAVR (21). Our findings just suggest that LAVR may serve as a potential valuable indicator of MR improvement following TAVR and provide clues for further investigation of this issue. The results was exploratory and should be interpreted as preliminary until the finding is externally validated. Future studies should incorporate more comprehensive echocardiographic assessment of all chamber dimensions to identify the optimal predictor of MR improvement.

This study used the color regurgitant jet area, a widely applied method for MR grading in clinical practice, which has certain limitations. It was recommended a multiparameter integrated approach, including effective regurgitant orifice area, vena contracta width, regurgitant volume and regurgitant fraction, to grade MR (22). However, this study showed that the proportion of AS patients with moderate-to-severe MR was 30.9%, and the rate of MR improvement after TAVR was 72.7%, which is similar to previously reported MR improvement rates (23), indicating that our findings remain clinically relevant. It should also be noted that the 2025 ESC/EACTS guideline criteria was applied retrospectively to patients treated earlier. However, given that the guideline updates primarily represent an expansion rather than a fundamental change in contraindications, this approach can avoid selection bias that might arise from differences in guideline versions across periods. Regarding the management of patients with severe AS and moderate-to-severe MR, the mainstream approach is a staged strategy, starting with TAVR followed by reassessment of MR during follow-up to guide decisions between medical therapy and transcatheter mitral valve repair (TMVr) (7). Our findings suggest that identification of clues for predicting whether MR improves after TAVR could avoid overly aggressive TMVr through appropriate intensification of guideline-directed medical therapy and extended follow-up. Furthermore, identifying overlapping factors and assessing left ventricular size can inform the likelihood of early MR improvement, thereby aiding in treatment response evaluation, post-TAVR medical therapy intensity, and follow-up planning. This study also integrates potential predictors into a pathophysiological framework of MR development, progression, and improvement, which supports comprehensive risk stratification based on left atrial and ventricular morphology combined with overlapping factors (including mitral valve etiology, LVEF, transvalvular pressure gradient, pulmonary artery pressure, AR, and CHD) (24). Future studies should explore and validate more valuable predictors within this mechanistic framework to guide clinical practice.

This study has several limitations. First, the sample size was small and derived from a single center. Despite the use of penalized firth regression and leave-one-out case-deletion analysis, the small sample size of this single-center study inevitably entails a risk of overfitting. For some variables in the comparative analysis, few positive cases precluded calculation of statistical effect values. Additionally, due to the limited sample size, further subgroup analyses based on the presence of PMR or other factors could not be performed. Moreover, because only patients undergoing TAVR (not SAVR) were included, the generalizability of our findings is limited. Second, the follow-up duration was relatively short, precluding assessment of the impact of residual MR or early MR improvement on long-term outcomes in this population. Finally, limited confounders were adjusted for in the regression analyses. Future studies with larger sample sizes in multiple centers, inclusion of more variables including more comprehensive echocardiographic parameters, and adjustment for additional confounders are needed to validate our findings.

In conclusion, MR severity improves after TAVR in most patients with severe AS and moderate-to-severe MR. The echocardiographic parameter LAVR, which integrates atrial and ventricular diameters, can provide clues for risk stratification regarding whether MR improves after TAVR. Further integration with other perioperative assessment data can help guide subsequent treatment strategies, observation duration, and intensity of medical therapy. Future studies should explore and validate more valuable predictors to inform clinical decision-making.

## Data Availability

The raw data supporting the conclusions of this article will be made available by the authors, without undue reservation.
