# Supplementary material for: The identification value of common echocardiography parameters for improvement of mitral regurgitation after transcatheter aortic valve replacement in patients with aortic stenosis
Source: Front Cardiovasc Med. 2026 Sep 21;13:1866427. doi: 10.3389/fcvm.2026.1866427 (PMC13635166; doi:10.3389/fcvm.2026.1866427)
Supplement: Supplementary file 1 [file Table1.docx]

| **Supplementary Table 1 Baseline comparisons between patients with complete follow-up and censored patients.** | | | | |
| --- | --- | --- | --- | --- |
|  | **Patients with**  **MR ≥ Grade 2 (N = 55)** | **Censored Patients (N = 9)** | **Patients with**  **complete follow-up (N = 46)** | ***P* value** |
| **Clinical Variables** |  |  |  |  |
| Age, years | 70.42 ± 7.34 | 70.67 ± 7.98 | 70.37 ± 7.30 | 0.913 |
| Male, n (%) | 24 (43.64) | 3 (33.33) | 21 (45.65) | 0.754 |
| BMI, kg/m^2^ | 23.10 ± 3.14 | 22.71 ± 4.88 | 23.17 ± 2.76 | 0.690 |
| Coronary artery disease, n (%) | 4 (7.27) | 1 (11.11) | 3 (6.52) | 0.522 |
| Diabetes, n (%) | 7 (12.73) | 0 (0.00) | 7 (15.22) | 0.480 |
| Hypertension, n (%) | 22 (40.00) | 3 (33.33) | 19 (41.30) | 0.941 |
| Prior MI, n (%) | 1 (1.82) | 0 (0.00) | 1 (2.17) | 1.000 |
| Prior PCI, n (%) | 2 (3.64) | 0 (0.00) | 2 (4.35) | 1.000 |
| Atrial fibrillation, n (%) | 10 (18.18) | 5 (55.56) | 5 (10.87) | **0.007** |
| Hemoglobin, g/L | 125.40 ± 19.34 | 130.00 ± 15.29 | 124.50 ± 20.06 | 0.440 |
| Ln (NT-proBNP) | 8.14 (6.79, 8.77) | 8.14 (7.38, 8.59) | 8.18 (6.76, 8.88) | 0.707 |
| SCr, μmol/L | 70.9 ± 30.7 | 68.11 ± 10.91 | 71.50 ± 33.27 | 0.765 |
| **Echocardiographic Variables before TAVR** |  |  |  |  |
| LVEDD, mm | 56.49 ± 10.47 | 53.22 ± 16.26 | 57.13 ± 9.06 | 0.310 |
| LVESD, mm | 39.91 ± 10.61 | 38.00 ± 15.32 | 40.28 ± 9.61 | 0.560 |
| IVST, mm | 11.18 ± 1.93 | 11.00 ± 2.00 | 11.22 ± 1.93 | 0.760 |
| LVPWT, mm | 10.33 ± 1.93 | 10.22 ± 1.79 | 10.35 ± 1.97 | 0.860 |
| RWT | 0.40 ± 0.10 | 0.43 ± 0.14 | 0.39 ± 0.10 | 0.245 |
| LAD, mm | 41.27 ± 6.7 | 42.33 ± 9.41 | 41.07 ± 6.22 | 0.611 |
| LAVR | 0.75 ± 0.16 | 0.83 ± 0.19 | 0.74 ± 0.16 | 0.129 |
| LVEF, % | 62.00 (48.50, 67.00) | 60.00 (58.00, 65.00) | 62.00 (48.00, 67.00) | 0.665 |
| Aortic valve velocity, cm/s | 472 ± 80 | 469.00 ± 61.46 | 472.43 ± 83.67 | 0.907 |
| Mean aortic gradient, mmHg | 51.4 ± 19.47 | 47.67 ± 16.29 | 52.20 ± 20.11 | 0.528 |
| MR area, cm^2^ | 6.00 (5.00, 8.00) | 6.00 (4.60, 6.40) | 6.05 (5.05, 8.00) | 0.412 |
| AR area, cm^2^ | 4.50 ± 4.25 | 4.20 ± 3.18 | 4.56 ± 4.46 | 0.818 |
| Moderate-to-severe AR, n (%) | 22 (40.00) | 3 (33.33) | 19 (41.30) | 0.941 |
| TR area, cm^2^ | 3.13 ± 2.76 | 4.67 ± 3.37 | 2.82 ± 2.56 | 0.067 |
| Pulmonary systolic artery pressure, mmHg | 39.69 ± 19.50 | 45.67 ± 17.65 | 38.52 ± 19.80 | 0.319 |
| The bold emphasis indicated that the difference was statistically significant. MR, mitral regurgitation; BMI, body mass index; MI, myocardial infarction; PCI, percutaneous coronary intervention; NT-proBNP, N-terminal pro-brain natriuretic peptide; SCr, serum creatinine; TAVR, transcatheter aortic valve replacement; LVEDD, left ventricular end-diastolic diameter; LVESD, left ventricular end-systolic diameter; IVST, interventricular septal thickness; LVPWT, left ventricular posterior wall thickness; RWT, relative wall thickness; LAD, left atrial diameter; LAVR, left atrial-to-ventricular ratio; LVEF, left ventricular ejection fraction; AR, aortic regurgitation; TR, tricuspid regurgitation. | | | | |
|  |  |  |  |  |
