# Supplementary material for: The identification value of common echocardiography parameters for improvement of mitral regurgitation after transcatheter aortic valve replacement in patients with aortic stenosis
Source: Front Cardiovasc Med. 2026 Sep 21;13:1866427. doi: 10.3389/fcvm.2026.1866427 (PMC13635166; doi:10.3389/fcvm.2026.1866427)
Supplement: Supplementary file 3 [file Table3.docx]

| **Supplementary Table 3 Sensitivity analysis of LAVR with MR improvement after TAVR.** | | | | |
| --- | --- | --- | --- | --- |
|  | **Crude Model** ^a^ | | **Adjusted Model** ^b^ | |
|  | **OR (95%CI**) | ***P*** **value** | **OR (95%CI)** | ***P* value** |
| **No. 27 excluded** ^c^ | 3.385 (1.181, 9.707) | **0.023** | 3.231 (1.042, 10.024) | **0.042** |
| **No. 44 excluded** | 3.588 (1.219, 10.556) | **0.020** | 3.290 (1.061, 10.199) | **0.039** |
| **No. 85 excluded** | 3.215 (1.144, 9.034) | **0.027** | 2.875 (0.981, 8.424) | 0.054 |
| **No. 104 excluded** | 4.647 (1.386, 15.580) | **0.013** | 3.728 (1.092, 12.727) | **0.036** |
| **No. 149 excluded** | 3.310 (1.220, 8.986) | **0.019** | 2.928 (1.026, 8.355) | **0.045** |
| **No. 233 excluded** | 3.331 (1.224, 9.067) | **0.019** | 2.994 (1.055, 8.498) | **0.039** |
| The bold emphasis indicated that the difference was statistically significant.  a. Crude model adjusted for none.  b. Adjusted Model adjusted for age, gender.  c. Sensitivity analyses were performed by sequentially excluding patients with no MR improvement in (n = 6) one at a time according to the Leave-One-Out case-deletion analysis, with the de-identified codes of these patients numbered accordingly.  LAVR, left atrial-to-ventricular ratio; MR, mitral regurgitation; TAVR, transcatheter aortic valve replacement; OR, odds ratio; 95%CI, 95% confidence interval. | | | | |
