# Supplementary material for: The identification value of common echocardiography parameters for improvement of mitral regurgitation after transcatheter aortic valve replacement in patients with aortic stenosis
Source: Front Cardiovasc Med. 2026 Sep 21;13:1866427. doi: 10.3389/fcvm.2026.1866427 (PMC13635166; doi:10.3389/fcvm.2026.1866427)
Supplement: Supplementary file 4 [file Table4.docx]

| **Supplementary Table 4 Sensitivity analysis of LAVR with MR improvement after TAVR after censored patients were counted as non-improvement.** | | |
| --- | --- | --- |
|  | **OR (95%CI)** | ***P* value** |
| **Crude Model** ^a^ | 2.506 (1.275, 4.925) | **0.008** |
| **Model 1** ^b^ | 2.375 (1.160, 4.860) | **0.018** |
| The bold emphasis indicated that the difference was statistically significant.  a. Crude model adjusted for none.  b. Model 1 adjusted for age, gender. LAVR, left atrial-to-ventricular ratio; MR, mitral regurgitation; TAVR, transcatheter aortic valve replacement; OR, odds ratio; 95%CI, 95% confidence interval. | | |
|  |  |  |
|  |  |  |
